# Current Cigarette Smoking Among Adults — United States, 2005–2013

**Published:** 2014-11-28

**Authors:** Ahmed Jamal, Israel T. Agaku, Erin O’Connor, Brian A. King, John B. Kenemer, Linda Neff

**Affiliations:** 1 Office on Smoking and Health, National Center for Chronic Disease Prevention and Health Promotion, CDC

Tobacco use is the leading cause of preventable disease and death in the United States, resulting in more than 480,000 premature deaths and $289 billion in direct health care expenditures and productivity losses each year (1). Despite progress over the past several decades, millions of adults still smoke cigarettes, the most commonly used tobacco product in the United States (2). To assess progress made toward the *Healthy People 2020* target of reducing the proportion of U.S. adults who smoke cigarettes to ≤12.0% (objective TU-1.1),* CDC used data from the 2013 National Health Interview Survey (NHIS) to provide updated national estimates of cigarette smoking prevalence among adults aged ≥18 years. Additionally, for the first time, estimates of cigarette smoking prevalence were assessed among lesbian, gay, or bisexual persons (LGB) using NHIS data. The proportion of U.S. adults who smoke cigarettes declined from 20.9% in 2005 to 17.8% in 2013, and the proportion of daily smokers declined from 16.9% to 13.7%. Among daily cigarette smokers, the proportion who smoked 20–29 cigarettes per day (CPD) declined from 34.9% to 29.3%, and the proportion who smoked ≥30 CPD declined from 12.7% to 7.1%. However, cigarette smoking remains particularly high among certain groups, including adults who are male, younger, multiracial or American Indian/Alaska Native, have less education, live below the federal poverty level, live in the South or Midwest, have a disability/limitation, or who are LGB. Proven population-based interventions, including tobacco price increases, comprehensive smoke-free policies in worksites and public places, high-impact anti-tobacco mass media campaigns, and easy access to smoking cessation assistance, are critical to reducing cigarette smoking and smoking-related disease and death among U.S. adults, particularly among subpopulations with the greatest burden (3).

NHIS is an annual, nationally representative, in-person survey of the noninstitutionalized U.S. civilian population. The NHIS core questionnaire is administered to a randomly selected adult in each sampled household. The 2013 NHIS included 34,557 respondents aged ≥18 years; the response rate was 61.2%. Current cigarette smokers were respondents who reported smoking ≥100 cigarettes during their lifetime and, at the time of interview, reported smoking every day or some days. The mean number of cigarettes smoked per day was calculated among daily smokers.

Data were adjusted for nonresponse and weighted to provide nationally representative estimates. Current cigarette smoking was assessed overall and by sex, age, race/ethnicity, education, poverty status,† U.S. Census region,§ and disability/limitation status.¶ Current smoking was also assessed by sexual orientation**; starting in 2013, sexual orientation questions were added to NHIS for the first time. Differences between groups were assessed using the chi-square test. Logistic regression was used to analyze trends during 2005–2013, and the Wald test was used to determine statistical significance (p<0.05).

Current cigarette smoking among U.S. adults declined from 20.9% (an estimated 45.1 million persons) in 2005 to 17.8% (42.1 million) in 2013 (p<0.05 for trend) (Table). In 2013, current cigarette smoking prevalence was higher among males (20.5%) than females (15.3%). Prevalence was highest among adults aged 25–44 years (20.1%) and lowest among those aged ≥65 years (8.8%). By race/ethnicity, prevalence was highest among adults reporting multiple races (26.8%) and among American Indians/Alaska Natives (26.1%), and lowest among non-Hispanic Asians (9.6%). By education (among adults aged ≥25 years), prevalence was highest among persons with a General Education Development (GED) certificate (41.4%) and lowest among those with a graduate degree (5.6%). Among groups by family income, prevalence was higher among persons living below the poverty level (29.2%) than those at or above this level (16.2%). By region, prevalence was highest in the Midwest (20.5%) and lowest in the West (13.6%). Adults who reported having a disability/limitation had a higher prevalence (23.0%) than those reporting no disability/limitation (17.0%). Cigarette smoking prevalence was higher among LGB adults (26.6%) than straight adults (17.6%). Among straight adults, males (20.3%) had a higher smoking prevalence than females (15.0%); however, among LGB adults, prevalence did not differ by sex (Figure 1).

Among all U.S. adults, the proportion of daily smokers declined from 16.9% to 13.7% during 2005–2013. Among current cigarette smokers, every day smoking decreased from 80.8% (36.5 million persons) in 2005 to 76.9% (32.4 million) in 2013 (p<0.05 for trend), and some day smoking increased from 19.2% (8.7 million) in 2005 to 23.1% (9.7 million) in 2013 (p<0.05 for trend). Among daily smokers, mean CPD declined from 16.7 in 2005 to 14.2 in 2013 (p<0.05 for trend). During 2005–2013, increases occurred in the proportion of daily smokers who smoked 1–9 CPD (16.4% to 23.3%) or 10–19 CPD (36.0% to 40.3%), whereas declines occurred among those who smoked 20–29 CPD (34.9% to 29.3%) or ≥30 CPD (12.7% to 7.1%) (p<0.05 for trend) (Figure 2).

## Discussion

During 2005–2013, declines occurred in the prevalence of cigarette smoking among U.S. adults and the proportion of daily smokers who smoked the heaviest (i.e., ≥30 CPD). Cigarette smoking prevalence was higher among certain subpopulations, including adults who are male, younger, multiracial or American Indian/Alaska Native, have less education, live below the federal poverty level, live in the South or Midwest, have a disability/limitation, or are LGB.

What is already known on this topic?

Tobacco use is the leading cause of preventable disease and death in the United States, resulting in more than 480,000 premature deaths and $289 billion in direct health care expenditures and productivity losses each year. Despite progress over the past several decades, millions of adults still smoke cigarettes, the most commonly used tobacco product in the United States.

What is added by this report?

Cigarette smoking among U.S. adults declined from 20.9% in 2005 (an estimated 45.1 million persons) to 17.8% in 2013 (42.1 million). Among smokers who smoke daily, the average number of cigarettes smoked per day declined from 16.7 in 2005 to 14.2 in 2013, and the proportions of daily smokers who smoked 20–29 or ≥30 cigarettes per day also declined. In 2013, cigarette smoking prevalence was higher among lesbian, gay, or bisexual adults (26.6%) than straight adults (17.6%).

What are the implications for public health practice?

These findings underscore the importance of continued implementation of effective public health interventions that can reduce smoking-related disparities and accelerate progress toward meeting the *Healthy People 2020* target to reduce the proportion of U.S. adults who smoke cigarettes to ≤12.0%. These evidence-based interventions include increasing the price of tobacco products, implementing and enforcing comprehensive smoke-free laws, warning about the dangers of tobacco use with high-impact antismoking media campaigns, and increasing access to help with quitting.

Observed disparities in smoking prevalence are consistent with previous studies (2). Differences by race/ethnicity might be partly explained by sociocultural influences and practices related to the acceptability of tobacco use (4). Differences by education might be partly attributable to variations in exposure and understanding of information about the health hazards of smoking (5). Responses to newly added questions on sexual orientation in the 2013 NHIS questionnaire†† revealed that LGB adults have higher cigarette smoking prevalence than their straight counterparts, which might be attributed to multiple factors, including, for example, greater stress due to social stigma and discrimination, and targeted marketing toward this population by the tobacco industry (6). These disparities underscore the importance of enhancing the implementation and reach of proven strategies to prevent and reduce tobacco use among these groups, as well as expanding questions on surveillance tools to better capture data on subpopulations with the greatest burden of tobacco use.

The 50th anniversary Surgeon General’s report on the health consequences of smoking concluded that disease and death from tobacco use are overwhelmingly caused by cigarettes and other combusted products, and that rapid elimination of their use will dramatically reduce this burden (1). Although the decline in overall cigarette smoking prevalence during 2005–2013 from 20.9% to 17.8% is encouraging, approximately 42.1 million adults still smoke cigarettes; this underscores the need for continued implementation of evidence-based interventions outlined in the World Health Organization MPOWER package.§§ These interventions include increasing the price of tobacco products, implementing and enforcing comprehensive smoke-free laws, warning about the dangers of tobacco use with high-impact antismoking media campaigns, and increasing access to help with quitting. Such population-based interventions have been shown to reduce population smoking prevalence (3). For example, in 2013, CDC’s national tobacco education campaign, *Tips from Former Smokers* (TIPS),¶¶ resulted in a 75% increase in average weekly calls to the national telephone quitline portal 1-800-QUIT-NOW, and the number of unique visitors to the Tips website ( http://www.cdc.gov/tips ) increased nearly 38-fold compared with the 4 weeks before the campaign (7). Additionally, the Surgeon General recently called for consideration of further strategies that could significantly accelerate the decline in smoking, including the reduction of nicotine content in cigarettes to nonaddictive levels and greater restrictions on sales, particularly at the local level, including bans on entire categories of tobacco products (1).

The findings in this report are subject to at least six limitations. First, cigarette smoking status was self-reported and not validated by biochemical testing; however, self-reported smoking status correlates highly with serum cotinine levels (8). Second, because NHIS does not include institutionalized populations and persons in the military, results are not generalizable to these groups. Third, the NHIS response rate of 61.2% might have resulted in nonresponse bias. Fourth, the questionnaire did not assess gender identity; the inclusion of transgender persons in addition to LGB persons would be expected to yield higher estimates of use among sexual minorities. Fifth, this report does not include estimates of cigar or other combustible tobacco use, which have generally not declined in recent years, and have even increased in some populations (1). Finally, these estimates might differ from those from other surveillance systems. These differences can be partially explained by varying survey methodologies, types of surveys administered, and definitions of current smoking; however, trends in prevalence are comparable across surveys (1).

Sustained, comprehensive state tobacco control programs funded at CDC-recommended levels accelerate progress towards reducing the health and economic burden of tobacco-related diseases in the United States (3). However, in 2014, despite combined revenue of more than $25 billion from settlement payments and tobacco excise taxes for all states, states will spend only $481.2 million (1.9%) on comprehensive tobacco control programs,*** representing <15% of the CDC-recommended level of funding for all states combined (3). Moreover, only two states (Alaska and North Dakota) currently fund tobacco control programs at CDC-recommended levels. Implementation of comprehensive tobacco control policies and programs can result in substantial reductions in tobacco-related morbidity and mortality and billions of dollars in savings from averted medical costs (3).

## Figures and Tables

**FIGURE 1:**
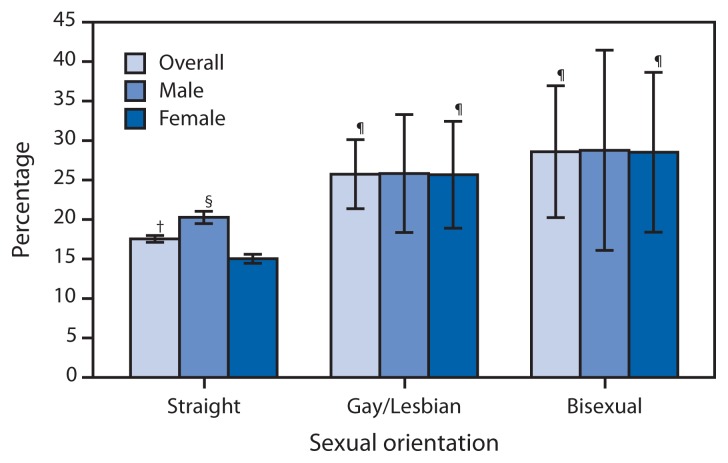
Current cigarette smoking among persons aged ≥18 years, by sex and sexual orientation^*^ — National Health Interview Survey, United States, 2013 ^*^ Sexual orientation was assessed with the following specified categories: “straight, that is, not gay” for men, “straight, that is, not gay or lesbian” for women, “gay” for men, “gay or lesbian” for women, and “bisexual” for either men or women. ^†^ 95% confidence interval. ^§^ Significantly different from straight females (p<0.05). ^¶^ Significantly different from straight (p<0.05).

**FIGURE 2:**
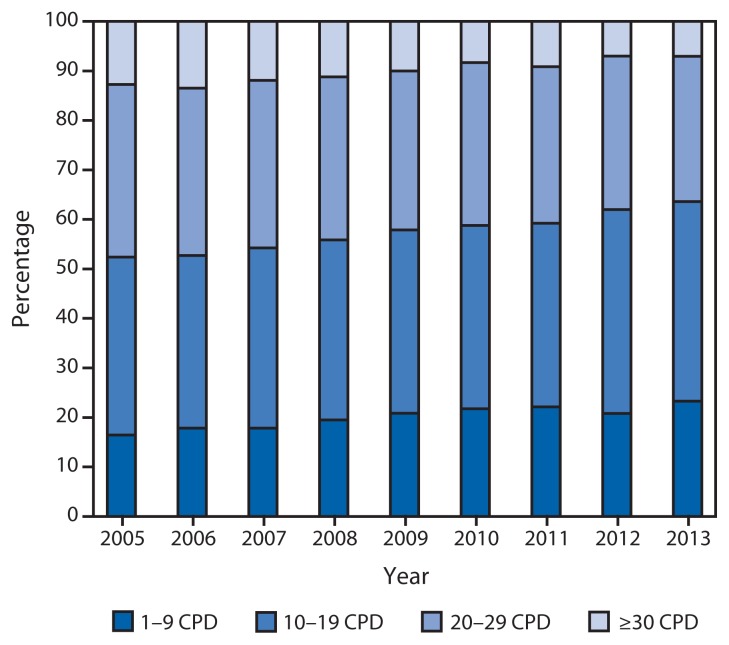
Percentage of daily smokers^*^ aged ≥18 years smoking 1–9, 10–19, 20–29, or ≥30 cigarettes per day (CPD), by year — National Health Interview Survey, United States, 2005–2013 ^*^ Persons who reported smoking ≥100 cigarettes during their lifetime and who, at the time of the survey, reported smoking cigarettes every day.

**TABLE:** Percentage of persons aged ≥18 years who were current cigarette smokers,* by selected characteristics — National Health Interview Survey, United States, 2005 and 2013

Characteristic	Men				Women				Total			
		
	2005 (n = 13,762)		2013 (n =15,440 )		2005 (n = 17,666)		2013 (n =19,117 )		2005 (N = 31,428)		2013 (N = 34,557)	
					
	%	(95% CI)	%	(95% CI)	%	(95% CI)	%	(95% CI)	%	(95% CI)	%	(95% CI)
**Overall**	**23.9**	**(22.9–24.8)**	**20.5** †	**(19.5–21.4)**	**18.1**	**(17.4–18.9)**	**15.3** †	**(14.6–16.1)**	**20.9**	**(20.3–21.5)**	**17.8** †	**(17.2–18.4)**
**Age group (yrs)**												
18–24	28.0	(25.0–31.1)	21.9†	(19.0–24.8)	20.7	(18.3–23.1)	15.4†	(12.9–17.9)	24.4	(22.4–26.4)	18.7†	(16.9–20.5)
25–44	26.8	(25.4–28.2)	23.3†	(21.7–24.9)	21.4	(20.2–22.6)	17.1†	(16.0–18.2)	24.1	(23.1–25.1)	20.1†	(19.1–21.1)
45–64	25.2	(23.7–26.7)	21.9†	(20.5–23.4)	18.8	(17.7–20.0)	18.1	(16.8–19.3)	21.9	(21.0–22.9)	19.9†	(19.0–20.9)
≥65	8.9	(7.6–10.2)	10.6	(9.2–11.9)	8.3	(7.3–9.3)	7.5	(6.5–8.4)	8.6	(7.8–9.3)	8.8	(8.0–9.7)
**Race/Ethnicity** §												
White	24.0	(22.8–25.2)	21.2†	(19.9–22.4)	20.0	(19.1–20.9)	17.8†	(16.8–18.8)	21.9	(21.1–22.7)	19.4†	(18.6–20.2)
Black	26.7	(23.9–29.4)	21.8†	(19.2–24.3)	17.3	(15.5–19.0)	15.4†	(13.7–17.0)	21.5	(19.8–23.1)	18.3†	(16.8–19.7)
Hispanic	21.1	(19.3–23.0)	17.3†	(15.3–19.2)	11.1	(9.8–12.4)	7.0†	(6.0–7.9)	16.2	(15.1–17.4)	12.1†	(11.0–13.2)
American Indian/ Alaska Native	37.5	(20.7–54.3)	32.1	(20.9–43.3)	26.8	(15.6–38.1)	22.0	(12.2–31.8)	32.0	(22.2–41.7)	26.1	(18.5–33.7)
Asian¶	20.6	(15.7–25.5)	15.1	(12.1–18.1)	6.1	(3.7–8.5)	4.8	(3.2–6.5)	13.3	(10.4–16.3)	9.6	(7.9–11.4)
Multiple race	26.1	(16.3–36.0)	29.1	(22.0–36.2)	23.5	(14.8–32.2)	24.8	(18.0–31.5)	24.8	(17.7–31.8)	26.8	(21.9–31.8)
**Education level** **												
0–12 years (no diploma)	29.5	(27.2–31.8)	30.6	(27.7–33.5)	21.9	(20.0–23.7)	18.0	(16.1–20.0)	25.5	(24.0–27.1)	24.2	(22.5–25.9)
8th grade or less	21.0	(17.7–24.3)	21.9	(17.3–26.5)	13.4	(11.1–15.6)	9.2	(6.8–11.6)	17.1	(15.1–19.0)	15.4	(12.8–17.9)
9–11th grade	36.8	(33.3–40.2)	40.0†	(36.0–44.0)	29.0	(26.1–31.8)	26.6	(23.2–29.9)	32.6	(30.4–34.9)	33.2	(30.6–35.8)
12th grade, no diploma	30.2	(23.5–36.9)	24.2	(18.3–30.1)	22.2	(16.9–27.5)	15.4	(11.1–19.8)	26.0	(21.8–30.2)	19.7	(16.0–23.5)
GED	47.5	(41.5–53.6)	42.9	(36.4–49.3)	38.8	(33.6–44.0)	39.7	(33.5–45.9)	43.2	(39.1–47.4)	41.4	(36.8–45.9)
High school diploma	28.8	(27.0–30.7)	26.7	(24.6–28.8)	20.7	(19.3–22.2)	17.6	(16.1–19.2)	24.6	(23.4–25.7)	22.0	(20.7–23.3)
Some college, no diploma	26.2	(24.0–28.4)	22.4†	(20.4–24.4)	21.1	(19.2–22.9)	19.5	(17.8–21.3)	23.5	(22.1–24.9)	20.9	(19.4–22.3)
Associate degree	26.1	(23.2–28.9)	17.8†	(15.5–20.2)	17.1	(15.0–19.3)	17.7	(15.5–20.0)	20.9	(19.2–22.6)	17.8†	(16.0–19.6)
Undergraduate degree	11.9	(10.5–13.3)	10.4†	(9.0–11.9)	9.6	(8.3–10.8)	7.9	(6.9–9.0)	10.7	(9.8–11.6)	9.1†	(8.3–10.0)
Graduate degree	6.9	(5.3–8.5)	5.7	(4.5–7.0)	7.4	(5.9–8.8)	5.5	(4.1–6.8)	7.1	(6.0–8.3)	5.6†	(4.7–6.5)
**Poverty status** ††												
At or above poverty level	23.7	(22.6–24.7)	18.7†	(17.7–19.7)	17.6	(16.8–18.5)	13.8†	(13.0–14.6)	20.6	(19.9–21.3)	16.2†	(15.6–16.8)
Below poverty level	34.3	(31.0–37.5)	33.8	(30.7–36.8)	26.9	(24.5–29.3)	25.8	(23.8–27.8)	29.9	(27.9–31.9)	29.2	(27.5–31.0)
Unspecified	21.2	(19.2–23.2)	19.9†	(17.2–22.5)	16.1	(14.8–17.5)	12.6†	(10.7–14.6)	18.4	(17.2–19.6)	16.0†	(14.3–17.7)
**U.S. Census region** §§												
Northeast	20.7	(18.6–22.9)	18.0†	(15.8–20.2)	17.9	(16.4–19.5)	15.8†	(14.0–17.7)	19.2	(17.8–20.6)	16.9†	(15.6–18.1)
Midwest	27.3	(25.3–29.3)	23.6†	(21.6–25.6)	21.3	(19.8–22.8)	17.4†	(15.5–19.3)	24.2	(23.0–25.3)	20.5†	(19.1–21.9)
South	25.3	(23.6–27.0)	22.7	(21.1–24.4)	18.5	(17.3–19.7)	16.2	(15.1–17.3)	21.8	(20.6–23.0)	19.2†	(18.2–20.3)
West	20.1	(18.3–21.9)	15.8†	(14.0–17.5)	13.9	(12.6–15.2)	11.5†	(10.3–12.7)	17.0	(16.0–18.0)	13.6†	(12.5–14.7)
**Disability/Limitation** ¶¶												
Yes	—***	—***	26.1	(23.6—28.7)	—***	—***	20.4	(18.5—22.3)	—***	—***	23.0	(21.4—24.5)
No	—***	—***	19.9	(18.6—21.2)	—***	—***	14.5	(13.5—15.5)	—***	—***	17.0	(16.2—17.7)
**Sexual orientation** †††												
Straight	—***	—***	20.3	(19.3–21.2)	—***	—***	15.0	(14.3–15.8)	—***	—***	17.6	(16.9–18.2)
Lesbian/Gay/Bisexual	—***	—***	26.4	(19.9–32.9)	—***	—***	26.7	(20.1–33.4)	—***	—***	26.6	(22.4–30.8)

**Abbreviations:** CI = confidence interval; GED = General Education Development certificate.

* Persons who reported smoking ≥100 cigarettes during their lifetime and who, at the time of interview, reported smoking every day or some days. Excludes 296 (2005) and 121 (2013) respondents whose smoking status was unknown.

† Denotes significant linear trend during 2005–2013 (p<0.05), adjusted for sex, age, and race/ethnicity as applicable.

§ Excludes 45 (2005) and 73 (2013) respondents of unknown race. Unless indicated otherwise, all racial/ethnic groups are non-Hispanic; Hispanics can be of any race.

¶ Does not include Native Hawaiians or Other Pacific Islanders.

** Among persons aged ≥25 years. Excludes 339 (2005) and 155 (2013) persons whose educational level was unknown.

†† Family income is reported by the family respondent who might or might not be the same as the sample adult respondent from whom smoking information is collected. 2005 estimates are based on reported family income and 2004 poverty thresholds published by the U.S. Census Bureau, and 2013 estimates are based on reported family income and 2012 poverty thresholds published by the U.S. Census Bureau.

§§ *Northeast:* Connecticut, Maine, Massachusetts, New Hampshire, New Jersey, New York, Pennsylvania, Rhode Island, and Vermont. *Midwest:* Illinois, Indiana, Iowa, Kansas, Michigan, Minnesota, Missouri, Nebraska, North Dakota, Ohio, South Dakota, and Wisconsin. *South:* Alabama, Arkansas, Delaware, District of Columbia, Florida, Georgia, Kentucky, Louisiana, Maryland, Mississippi, North Carolina, Oklahoma, South Carolina, Tennessee, Texas, Virginia, and West Virginia. *West:* Alaska, Arizona, California, Colorado, Hawaii, Idaho, Montana, Nevada, New Mexico, Oregon, Utah, Washington, and Wyoming.

¶¶ Disability/limitation was defined based on self-reported presence of selected impairments, including vision, hearing, cognition, and movement. Limitations in performing activities of daily living was defined based on response to the question, “Because of a physical, mental, or emotional problem, does [person] need the help of other persons with personal care needs, such as eating, bathing, dressing, or getting around inside this home?” Limitations in performing instrumental activities of daily living was defined based on response to the question, “Because of a physical, mental, or emotional problem, does [person] need the help of other persons in handling routine needs, such as everyday household chores, doing necessary business, shopping, or getting around for other purposes?” Any disability/limitation was defined as a “yes” response pertaining to at least one of the disabilities/limitations listed (i.e., vision, hearing, cognition, movement, activities of daily living, or instrumental activities of daily living). In 2013, the American Community Survey disability questions were asked of a random half of families at the end of the family interview, with proxy reporting permitted for family members not present during the interview. For population estimates, the sample adult weight was doubled to account for the half of respondents for whom the disability questions were not asked.

*** Questions for pertaining to disabilities/limitations and sexual orientation were not included in the 2005 National Health Interview Survey.

††† Response option were “straight, that is, not gay” for men, “straight, that is, not gay or lesbian” for women, “gay” for men, “gay or lesbian” for women, and “bisexual” for either men or women.

## Footnotes

* Additional information available at https://www.healthypeople.gov/2020/topics-objectives/topic/tobacco-use/objectives.

† Based on reported family income. 2005 estimates are based on reported family income and 2004 poverty thresholds published by the U.S. Census Bureau, and 2013 estimates are based on reported family income and 2012 poverty thresholds published by the U.S. Census Bureau.

§ *Northeast:* Connecticut, Maine, Massachusetts, New Hampshire, New Jersey, New York, Pennsylvania, Rhode Island, and Vermont. *Midwest:* Illinois, Indiana, Iowa, Kansas, Michigan, Minnesota, Missouri, Nebraska, North Dakota, Ohio, South Dakota, and Wisconsin. *South:* Alabama, Arkansas, Delaware, District of Columbia, Florida, Georgia, Kentucky, Louisiana, Maryland, Mississippi, North Carolina, Oklahoma, South Carolina, Tennessee, Texas, Virginia, and West Virginia. *West:* Alaska, Arizona, California, Colorado, Hawaii, Idaho, Montana, Nevada, New Mexico, Oregon, Utah, Washington, and Wyoming.

¶ Disability/limitation was defined based on self-reported presence of selected impairments, including vision, hearing, cognition, and movement. Limitations in performing activities of daily living was defined based on response to the question, “Because of a physical, mental, or emotional problem, does [person] need the help of other persons with personal care needs, such as eating, bathing, dressing, or getting around inside this home?” Limitations in performing instrumental activities of daily living was defined based on response to the question, “Because of a physical, mental, or emotional problem, does [person] need the help of other persons in handling routine needs, such as everyday household chores, doing necessary business, shopping, or getting around for other purposes?” Any disability/limitation was defined as a “yes” response pertaining to at least one of the disabilities/limitations listed (i.e., vision, hearing, cognition, movement, activities of daily living, or instrumental activities of daily living). In 2013, the American Community Survey disability questions were asked of a random half of families at the end of the family interview, with proxy reporting permitted for family members not present during the interview. For population estimates, the sample adult weight was doubled to account for the half of respondents for whom the disability questions were not asked.

** For the question used to determine sexual orientation, response options were “straight, that is, not gay” for men, “straight, that is, not gay or lesbian” for women, “gay” for men, “gay or lesbian” for women, and “bisexual” for either men or women.

†† Additional information available at http://www.cdc.gov/nchs/data/nhsr/nhsr077.pdf.

§§ Additional information available at http://www.who.int/tobacco/mpower/mpower_report_full_2008.pdf.

¶¶ Additional information available at http://www.cdc.gov/tobacco/campaign/tips.

*** Information available at http://www.tobaccofreekids.org/what_we_do/state_local/tobacco_settlement.
